# Applications of Machine Learning for the Classification of Porcine Reproductive and Respiratory Syndrome Virus Sublineages Using Amino Acid Scores of ORF5 Gene

**DOI:** 10.3389/fvets.2021.683134

**Published:** 2021-07-23

**Authors:** Jeonghoon Kim, Kyuyoung Lee, Ruwini Rupasinghe, Shahbaz Rezaei, Beatriz Martínez-López, Xin Liu

**Affiliations:** ^1^Department of Mathematics, University of California, Davis, Davis, CA, United States; ^2^Department of Medicine and Epidemiology, Center for Animal Disease Modeling and Surveillance (CADMS), School of Veterinary Medicine, University of California, Davis, Davis, CA, United States; ^3^Department of Computer Science, University of California, Davis, Davis, CA, United States

**Keywords:** artificial intelligence, random forest, k-nearest neighbor, support vector machine, swine health, phylogenetic tree, multilayer perceptron, classification

## Abstract

Porcine reproductive and respiratory syndrome is an infectious disease of pigs caused by PRRS virus (PRRSV). A modified live-attenuated vaccine has been widely used to control the spread of PRRSV and the classification of field strains is a key for a successful control and prevention. Restriction fragment length polymorphism targeting the Open reading frame 5 (ORF5) genes is widely used to classify PRRSV strains but showed unstable accuracy. Phylogenetic analysis is a powerful tool for PRRSV classification with consistent accuracy but it demands large computational power as the number of sequences gets increased. Our study aimed to apply four machine learning (ML) algorithms, random forest, k-nearest neighbor, support vector machine and multilayer perceptron, to classify field PRRSV strains into four clades using amino acid scores based on ORF5 gene sequence. Our study used amino acid sequences of ORF5 gene in 1931 field PRRSV strains collected in the US from 2012 to 2020. Phylogenetic analysis was used to labels field PRRSV strains into one of four clades: Lineage 5 or three clades in Linage 1. We measured accuracy and time consumption of classification using four ML approaches by different size of gene sequences. We found that all four ML algorithms classify a large number of field strains in a very short time (<2.5 s) with very high accuracy (>0.99 Area under curve of the Receiver of operating characteristics curve). Furthermore, the random forest approach detects a total of 4 key amino acid positions for the classification of field PRRSV strains into four clades. Our finding will provide an insightful idea to develop a rapid and accurate classification model using genetic information, which also enables us to handle large genome datasets in real time or semi-real time for data-driven decision-making and more timely surveillance.

## Introduction

Porcine reproductive and respiratory syndrome is one of the most important infectious diseases of pigs caused by PRRS virus (PRRSV), an enveloped RNA virus in the genus arterivirus. The virus causes reproductive failures in sows and respiratory disease in pigs of all ages, resulting in significant economic losses in the swine industry worldwide including in the United States of America (US), in which the annual losses have been estimated at $664 million (1). PRRSV strains diverged into multiple lineages globally and two major genotypes were reported in distinct geographical regions: Type 1 PRRSV in Europe and type 2 in North America (2). A modified live-attenuated vaccine (MLV) developed for type 2 PRRSV (e.g., Ingelvac PRRS® MLV by Boehringer Ingelheim Vetmedica, Inc. for lineage 5) has been widely used to control PRRSV in the US Porcine industry for more than 20 years (3). However, the generation and spread of novel strains and/or the virulence reversion of vaccine strains (4) have an impact on the efficacy of MLV and consequent spread of PRRSV type 2 in the US swine population. Consequently, the classification of field PRRSV strains played an important role of successful control and prevention measures of PRRSV type 2 in the US using MLV, especially for monitoring the effectiveness of vaccination as well as the development of new vaccine such as vaccine lineage selection. (e.g., Prevacent® by Elanco Inc. for lineage 1, PrimePac™ by Merck, Inc. for lineage 7, Fostera® by Zoetis and Ingelvac ATP by Boehringer Ingelheim Vetmedica, Inc. for lineage 8) (5).

The PRRSV genome consists of 10 open reading frame (ORF) genes (1a, 1b, 2a, 2b, 3, 4, 5, 5a, 6, 7), and the ORF5 gene encodes the GP5 protein; a hypervariable and immunogenic domain of PRRSV. The genetic information of the ORF5 gene in PRRSV is a key target to classify field PRSSV strains and evaluate the cross-protection induced by MLV (6, 7). Restriction fragment length polymorphism (RFLP) typing has been widely used to classify field strains due to relatively low experimental cost and short time consumption (8). However, current experimental verification of RFLP typing casted doubt on the stability and accuracy in PRSSV classification considering continuous mutation in the ORF5 gene even among field PRRSV strains with very close genetic relatedness (9). Corresponding to the current use of viral genome sequencing and open-source genomic data repositories, phylogenetic analysis has been increasingly employed to classify PRRSV strains due to consistent accuracy. However, phylogenetic analysis posed a challenge to estimate the phylogeny with the large number of genetic sequences because of the exponential increase of computational power for the calculation of likelihoods of possible combination phylogenies.

Machine learning (ML) has been widely used for classification and prediction in computer vision and natural language processing (10). ML is preferred for highly complex classification including multi- dimension and multi-class dataset rather than the regression model because ML has a strength to find the best-fit decision boundaries among discrete values and output class labels. A variety of ML algorithms have been developed for classification. Specifically, four ML algorithms, random forest (RF), support vector machine (SVM), k-nearest neighbors (KNN), and multilayer perceptron (MLP) are widely applied because of high accuracy, applicability, and adaptability (11, 12). Despite of the great potential, ML has not been easily applied for the classification using genetic information such as DNA, RNA, or amino acid sequences. Genome data are coded in long strings of alphabetic letters describing unique biochemical components [e.g., Adenine (A), Guanine (G), Cytosine (C), and Thymine (T)]. Therefore, simple transformation of the genome data in the numeric form possibly leads to significant information loss. Atchley et al. (13) presented the approach to transform the amino acid sequence into five numerical scores describing physicochemical properties (e.g., polarity, secondary structure, molecular volume, codon diversity, and electrostatic charge) (13). The amino acid score provided an availability to use ML algorithms for prediction and classification of phenotypic characteristics based on genetic information (14).

The present study aimed to classify US field PRRSV stains into four clades using four ML approaches based on amino acid scores of ORF5 gene. In second, we will also detect key amino acid positions for the classification. To the best of our knowledge, our work is the first attempt to apply four ML algorithms for classification of field PRRSV strains. Our study will provide an insightful idea to develop a rapid and accurate classification model using genetic information of infectious pathogens, which also enables us to handle large datasets in real time or semi-real time for data-driven decision-making and more timely surveillance or intervention strategies.

## Materials and Methods

### Data Collection and Phylogenetic Analysis

We collected ORF5 genome sequences and RFLP types of 1931 field PRRSV strains isolated from 328 porcine premises managed by two US pork production systems from 2012 to 2020. Multiple sequence comparison by log-expectation was used to align ORF5 nucleotide sequences on AliView (Version 1.26) (15). Homogeneity over alignment was evaluated, and common almost-pure-gap sites and the last three sites of stop codon were removed. The best-fit nucleotide substitution model and the among site rate variation were determined by ModelFinder (16) based on Bayesian information criterion (BIC). The best-fit model including among site rate variation and the partition scheme corresponding to codon positions were used to estimate the phylogeny of 1931 ORF5 gene through the maximum likelihood approach on IQ-TREE multicore (Version 2.1.1) in CIPRES Science Gateway (Version 3.3) (17). Bootstrap values were assessed using the ultrafast bootstrap approximation method with 5,000 replicates. The phylogenetic tree was visualized by the interactive Tree of Life (iTOL) tool (18). All 1931 ORF5 gene sequences of US field PRRSV samples were labeled into one of four clades: Lineage 5 (L5 clade) or three clades in Linage 1 (L1A clade: Sublineage 1.5, L1B clade: Sublineage 1.6, and L1C clade: Sublineages 1.7, 1.8, and 1.9) based on the topology of phylogeny with high bootstrap values (>95%) according to the global PRRSV classification systems (19).

### Data Transformation for the Classification Using Four ML Algorithms

**Figure 1:**
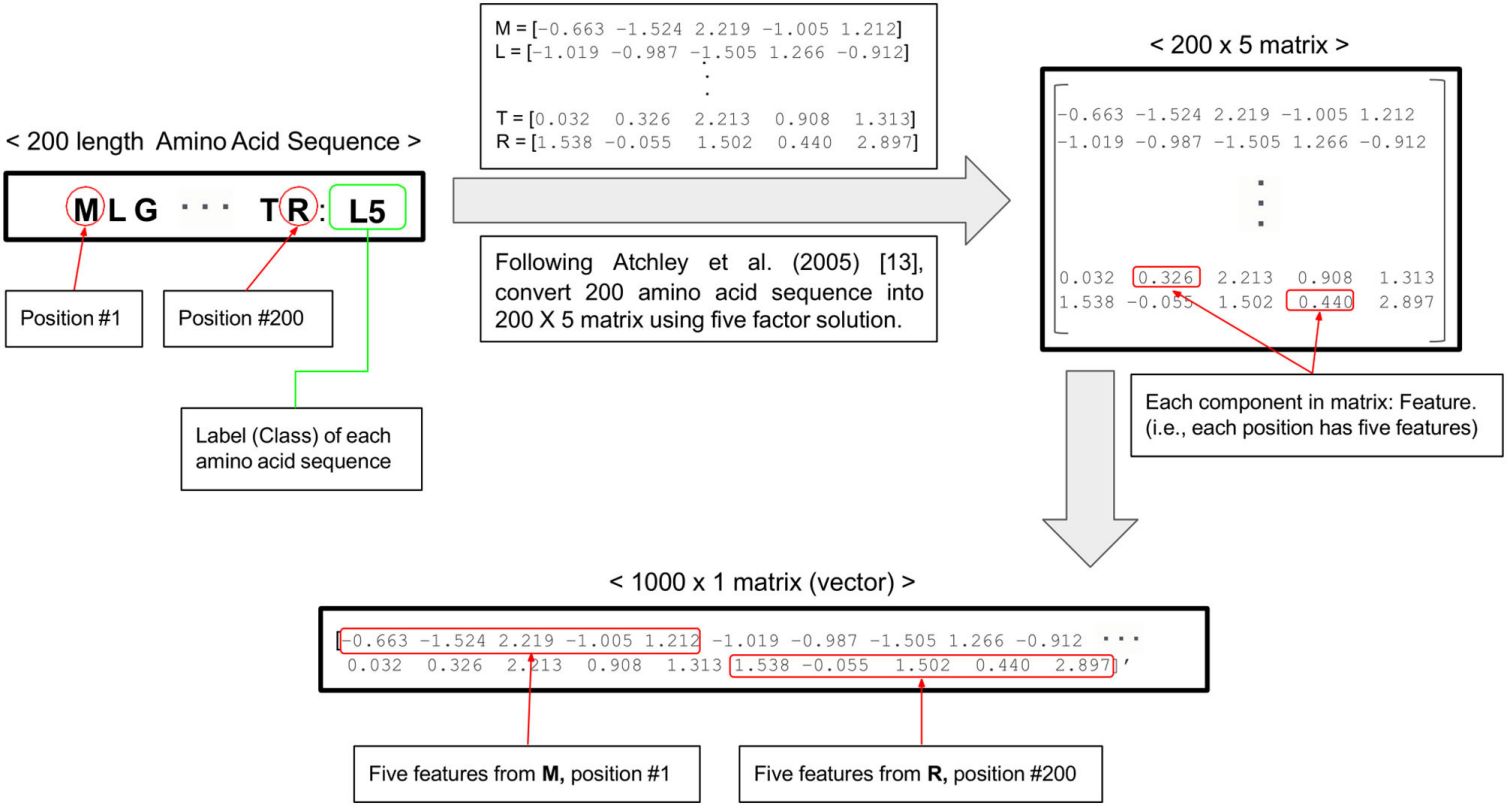
Flow chart of data preprocessing from one amino acid sequence into five numeric scores.

The nucleotide alignment of ORF5 gene of 1931 PRRSV samples with 600 nucleotide base-pairs were converted into the alignment of amino acid sequences with 200 amino acids long based on genetic code using AliView. Each amino acid sequence transformed to a 5 × 1 vector including five numerical scores of physiochemical amino acid properties (e.g., polarity, secondary structure, molecular volume, codon diversity, and electrostatic charge) (13). The 200 (amino acid sequences) × 5 (numeric score) matrix of each PRRSV ORF5 gene were changed to the 1,000 × 1 matrix for technical convenience. Finally, we built 1931 matrices with 1,000 features (1,000 × 1) of PRRSV ORF5 gene sequence for the classification into four clades by four machine learning algorithms (Figure 1). The distribution of 1,000 multivariate features and their clusters by four clades were visualized in two-dimension by principal component analysis (PCA).

### Machine Learning Algorithms

Our study used four ML algorithms, random forest (RF), support vector machine (SVM), k-nearest neighbors (KNN), and multilayer perceptron (MLP), to classify field PRRSV strains among four clades based on the five amino acid score of ORF5 gene. Additionally, information on theoretical time complexity using big-O-notation were provided.

#### Random Forest (RF)

RF is a supervised ML algorithm which is used for both classification and regression (20). RF creates several decision trees using training samples and obtains a class prediction from each of the decision trees, and it outputs the final class by majority-voting at inference time to achieve high accuracy. RF also provides the importance scores in classification for all features (i.e., 1,000 attributes for each sequence), which indicates the importance of each feature for RF classification, by using entropy and information gain. Importance score is the value between 0 and 1; the greater this value is, the more important in classification corresponding feature is. The information of importance scores in classification for all features were used feature selection in experiments. Training time complexity of RF is O(n^*^log(n)^*^d^*^k) where k is number of decision tree, n is number of samples, and d is number of dimension (features) (21).

#### Support Vector Machine (SVM)

SVM is one of supervised ML algorithm finding decision boundaries, so-called “a hyperplane,” for the classification of data points in an *N*-dimensional space (e.g., N variable) (22). SVM determines one hyperplane with the maximum margin among many possible hyperplanes based on the maximum distance between the hyperplane and data points in both classes. For data with non-linear and complex feature, a non-linear kernel (e.g., polynomial and Radial Based Function) is often used to map the input into a high dimensional feature space. However, the present study achieved a good classifier without using any non-linear kernel. Training time complexity for SVM is between O(n) and O(n^2.3^) where n is number of the training sample (23).

#### k-nearest Neighbors (KNN)

KNN is one of supervised ML algorithm using the proximity of data points for classification based on the assumption, “Similar things are near to each other” (24). Proximity is generally defined by a distance function. The distance function finds *k* neighbor data points in the training set nearest to the input. Then, the majority-vote is performed over the label of the k data points to predict the label of the input. Therefore, higher value of *k* often makes the model less sensitive to noise at the cost of more computation. The present study used Euclidean distance as a distance function. The *k* is a hyper-parameter of our KNN algorithm. Our data achieved good performance when *k* = 5. Training time complexity of KNN is O(1), and prediction time complexity is O(k^*^n^*^d) where n, d are the numbers of training samples and dimensions (features), respectively.

#### Multilayer Perceptron (MLP)

MLP is one of the fundamental feedforward neural network architectures used for classification (25). MLP uses one or more hidden layers consist of many nodes between input and output layers. The MLP architecture takes input data, returns some outputs and improves the accuracy by repeating three steps: each node (1) takes a weighted sum of its inputs on the connected nodes in the previous layer, (2) performs a non-linear operation (called activation function), and (3) passes the output to some connected nodes in the right next layer. The present study used back propagation for the training of MLP to obtain optimal weights and bias (26). To reach an approximated solution, tough time complexity of MLP is O(E^*^n^*^d^*^N) where E is number of epochs, n is number of training samples, d is number of dimensions (features), and N is the number of neurons (nodes) in the architecture.

### Evaluation of Classification Into Four Clades Using Four ML Algorithms by Accuracy and Time Consumption

#### RF Classification and Detection of Key Amino Acid Positions

We employed RF to classify 1931 US field PRRSV samples into four clades based on the matrix of 1,000 features. RF returned the importance scores for all 1,000 features. Importance score for one amino acid position was the sum of five importance scores for five features describing physiochemical properties of one amino acid in the position. For example, the importance score of the amino acid position #1 were the sum of five importance scores of the feature #1 to #5 in the matrix of 1,000 features because feature #1 to #5 are the five physiochemical amino acid properties of features of the position #1 amino acid.

#### Classification Accuracy and Time Consumption of Four ML Algorithms by the Number of Amino Acid Sequences

We evaluated the accuracy and time consumption for field PRRSV classification into four clades using four ML algorithms (RF, SVM, KNN, and MLP). We firstly measured the accuracy and time consumption of four ML algorithms using 200 amino acid positions. To evaluate how the number of amino acid sequences affected the performance of four ML algorithms, we measured the accuracy and time consumption of four ML algorithms using one amino acid position with the highest RF importance score and sequentially added amino acids from the position with the second highest RF importance score. The 10-fold cross-validation was assigned, and training and test data were randomly split in each run. Each experiment conducted 100 different runs, and for each run accuracy and time consumption including training and test were outputted. The area under curve (AUC) of the receiver of operating characteristics curve (ROC) (27) was used to evaluate the accuracy of classification as well as precision, recall, and f1-score (28). Precision, recall, and f1-score are outputted for each class and class-wise weighted averaged results were provided to take class imbalance into account. All experiments were conducted on Python (Version 3.7.6).

#### Training Details With Parameter Tuning

There were hyper parameters for each ML algorithm that could affect its accuracies and performances. Hyperparameters were tuned for optimal performance. The training details for each ML algorithm experiments were as follows: (1) RF experiments. One hundred trees were used, and max of depth was not assigned, which means nodes were expanded until all leaves were pure or all leaves contained less than minimum sample split samples (fixed as 2 in our experiments). (2) SVM experiments. Linear kernel, a main hyperparameter, was adopted and 0.0001 was used as a learning rate, and also max iteration was not determined. (3) KNN experiments. *k* = 5 was selected after comparison with other integers. (4) MLP experiments. One hidden layer with 128 nodes followed by ReLU activation function was used, and the softmax function was used for the output layer.

## Results

### Phylogenetic Analysis for Labeling and RF Classification and Detection of Key Amino Acid Positions

**Figure 2:**
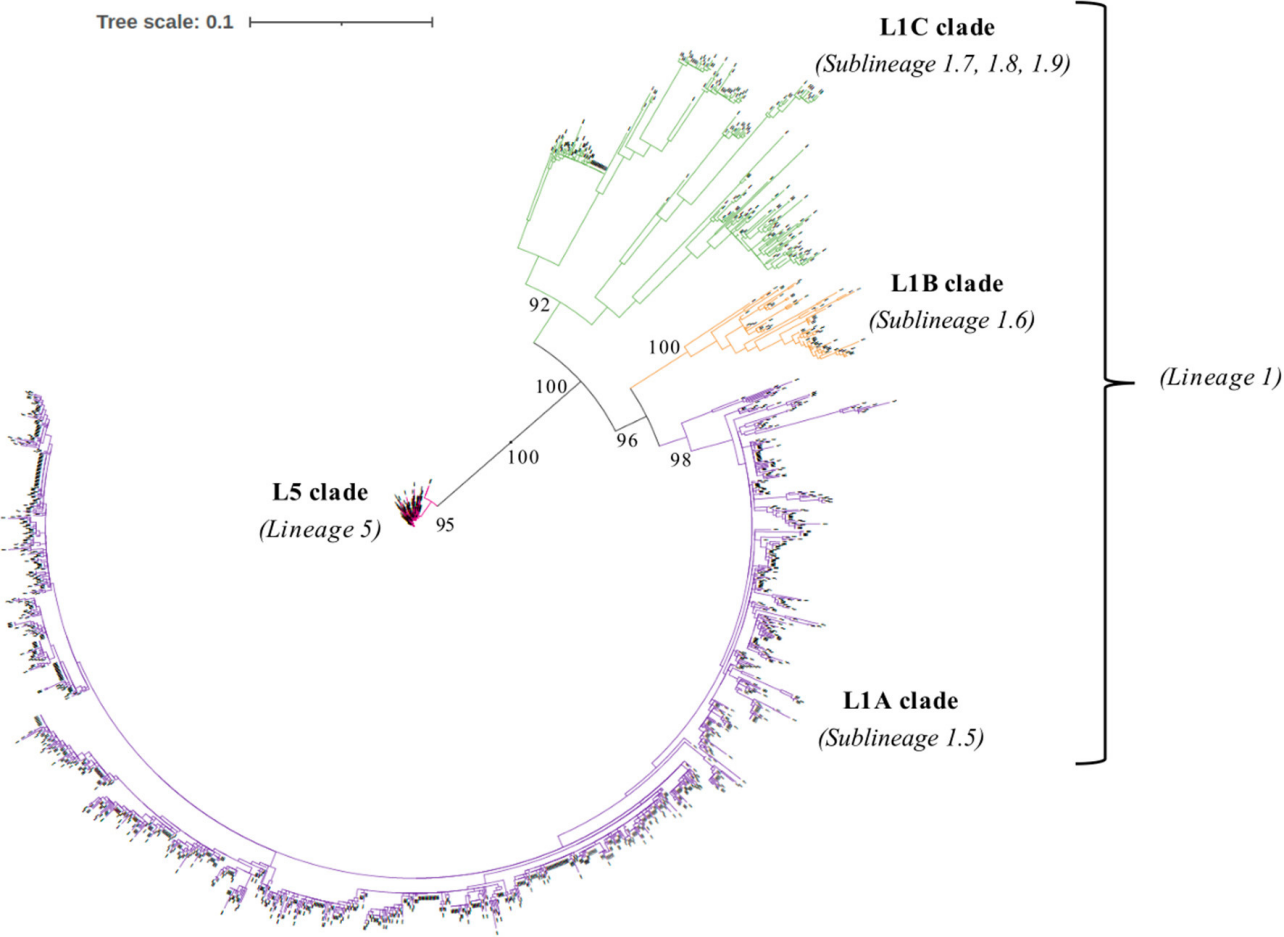
The phylogeny of 1931 field Porcine reproductive and respiratory syndrome virus (PRRSV) field strains estimated by maximum likelihood approach based on the nucleotide sequences of open reading frame 5 (ORF5) gene.

**Figure 3:**
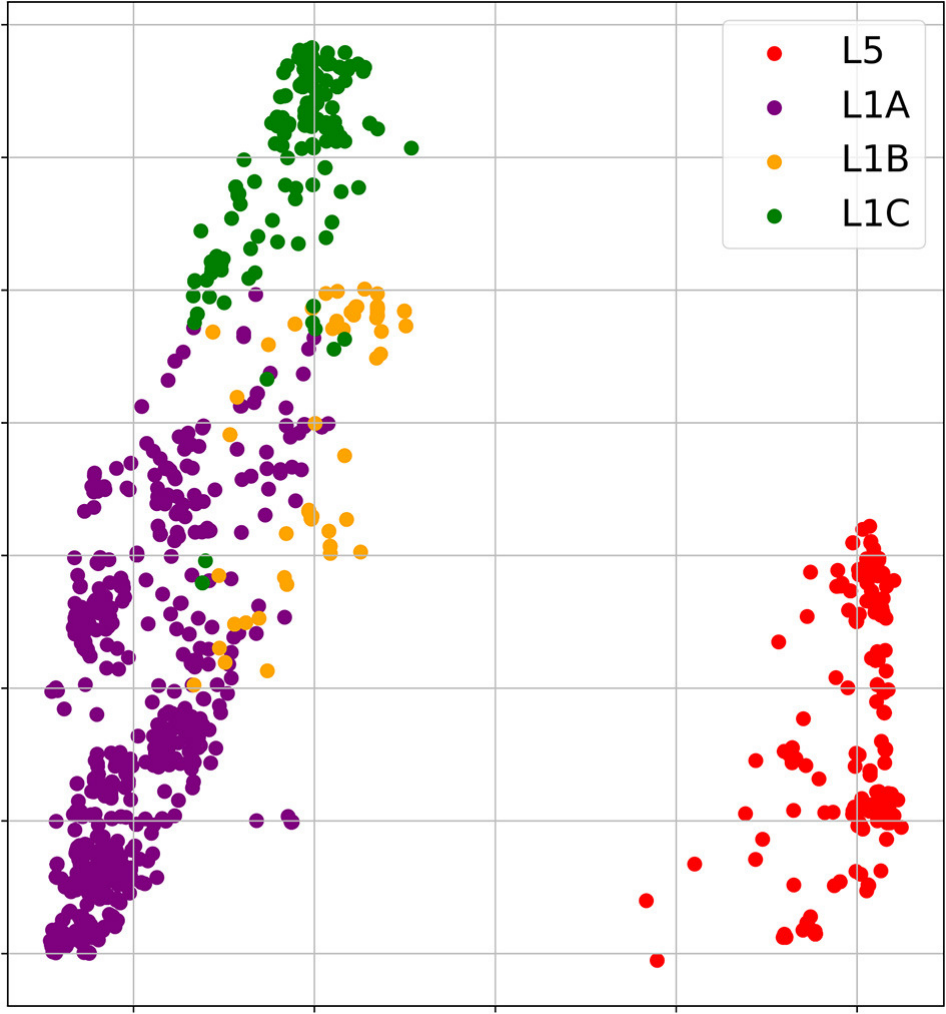
Principal component analysis (PCA) visualizations of 1,000 amino acid scores of 1931 field PRRSV strains. [Red: L5 clade, purple: L1A clade, yellow: L1B clade & green: L1C clade].

The phylogenetic analysis of 1931 field PRRSV strains using ORF5 gene showed two distinct clades involving 438 L5 clade (22.6%) and 1498 L1 clade (77.4%) (Figure 2). Field PRRSV strains in L1 clade were further classified into one of three Sublineages (L1A clade: Sublineage 1.5, L1B clade: Sublineage 1.6, and L1C clade: Sublineages 1.7, 1.8, and 1.9). PCA visualization of the classification presented the clear margin between clusters of L1 and L5 clades (Figure 3). However, we observed contiguous margins for the classification among L1A, L1B, and L1C clades.

**Figure 4:**
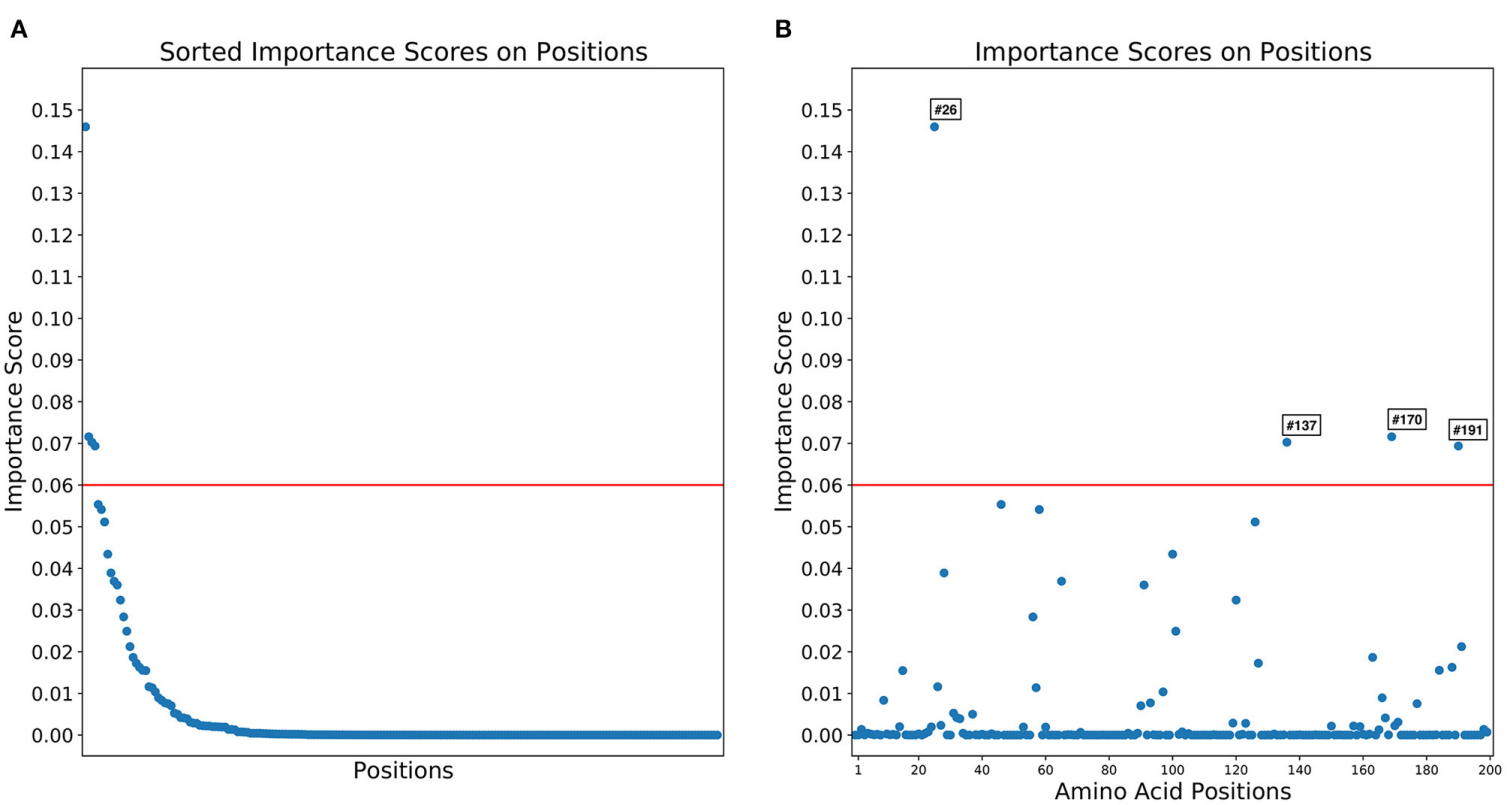
Distributions of importance scores for random forest classification of 1931 field PRRSV strains between four clades based on 200 amino acid positions. **(A)** The distribution importance scores in descending order. **(B)** Importance scores by the amino acid sequences.

Importance scores of RF classification in each amino acid position were outputted by RF. RF found that highly right-skewed distribution of importance score in amino acid positions (Figure 4) and four amino acid positions showed notably higher importance scores than other position (> 0.06) [26th, 170th, 137th, and 191 st] (Table 1). The 26th position showed significant heterogeneity of amino acid sequences between L5 (A: 98.4%) and L1A (V: 98.3%) clades. The amino acid sequence of L5 (E: 99.5%) and L1A (E: 98.1%) clades in the 170th position was also heterogenous comparing to L1B (N: 100%) and L1C (G: 100%) clades. The 137th amino acid position was a key site to classify between L5 (A: 99.8%) and three L1 (S: 100%) clades (Table 1).

**Table 1:** Top 4 key amino acid positions in open reading frame 5 gene with the highest random forest importance scores for the 1931 field PRRS strain classification [Importance score > 0.06].

**Amino acid position**	**Importance score**	**Amino acid in L5 clade (%)** **(*n* = 438)**	**Amino acids in L1A clade (%)** **(*n* = 1,225)**	**Amino acids in L1B clade (%)** **(*n* = 69)**	**Amino acids in L1C clade (%)** **(*n* = 199)**
26	0.145	A (98.4) V (0.9) T (0.7)	V (98.3) I (1.6) A (0.2)	V (94.2) A (5.8)	A (96.5) D (0.5) V (3.0)
170	0.071	E (99.5) G (0.5)	E (98.1) G (1.6) K (0.3)	N (100)	G (100)
137	0.070	A (99.8) X (0.2)^*^	S (100%)	S (100%)	S (100%)
191	0.063	R (99.5) Q (0.2) X (0.2)^*^	K (99.8) X (0.2)	K (100)	R (64.8) K (34.7) S (0.5)

* *X is sequencing error*.

RFLP analysis classified our 1931 field PRRSV strains into 43 types (Supplementary Table 1). The RFLP type classified all 1931 PRRSV strains into either L5 (7 types) or L1 (36 types) clades correctly. Almost all strains in L5 clades were classified into 2-5-2 RFLP type (93.6%, 410/438). PRRSV strains in one of three L1 clades were classified into either 1-8-4 (59.3%, 888/1498), 1-7-4 (19.9% 298/1498), or 1-4-4 (6.8% 102/1498) RFLP type. However, for the classification of three L1 clades, field PRRSV strains in 9 RFLP types (1-3-2, 1-3-4, 1-4-3, 1-4-4, 1-7-4, 1-8-3, 1-8-4, 1-12-4, and 1-16-4) belonged to two L1 clades at the same time (L1A & L1C clades or L1B & L1C clades) (Supplementary Table 1).

### The Accuracy and Time Consumption of Classification Using Four ML Algorithms by the Number of Amino Acid Sites

**Figure 5:**
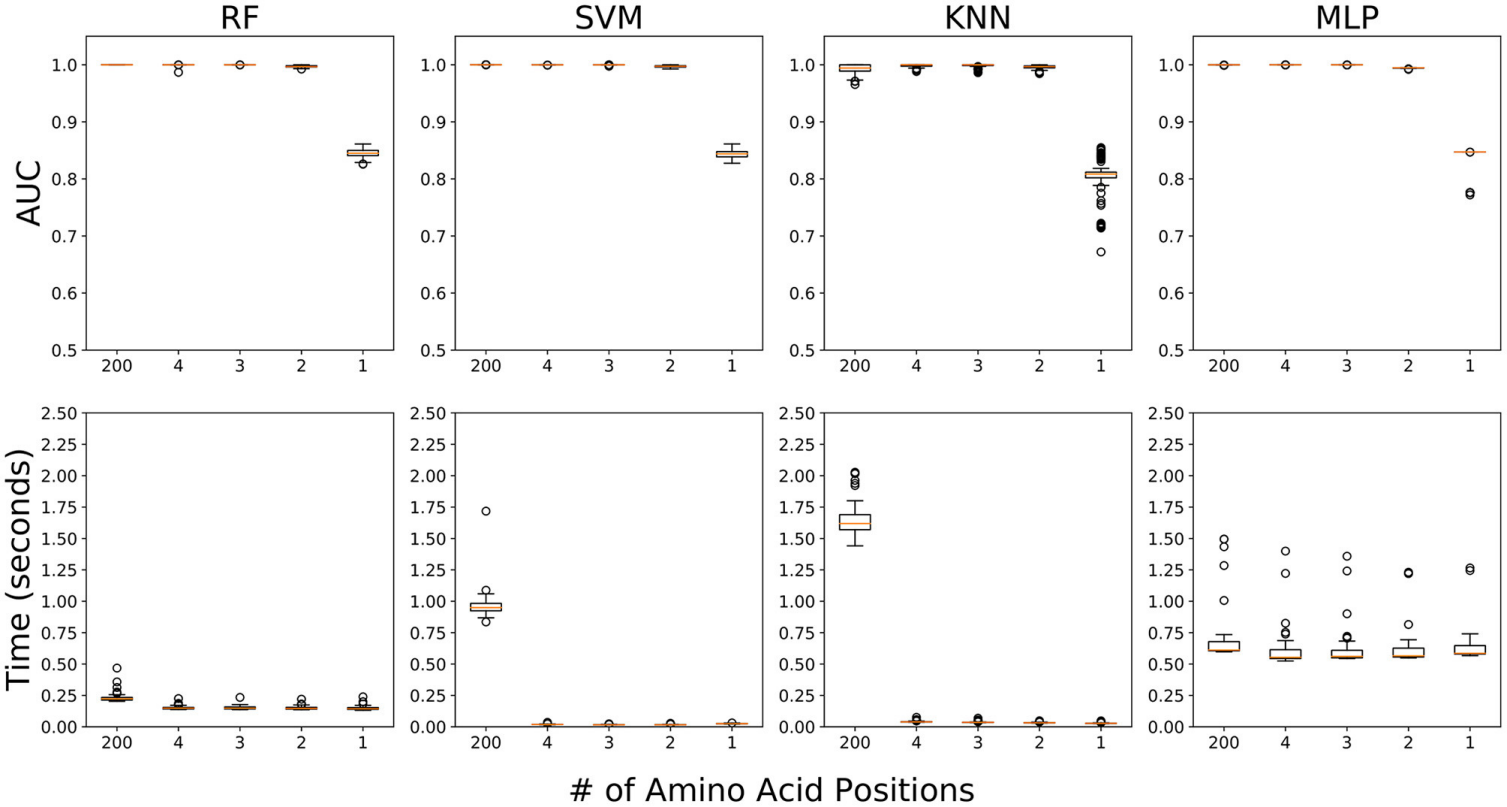
Accuracy and time consumption of four machine learning algorithms in five experiments for PRRSV classification. First experiment fully includes 200 amino acid positions and other four experiments sequentially involved the top 4 amino acid positions [26th, 170th, 137th, and 191st] by their importance score. Top: area under the curve (AUC) values. Bottom: time consumption (seconds). Orange lines are mean values over 100 runs.

We performed five ML experiments included 1) the data fully utilizing 200 amino acid positions, and 2) the four data sequentially adding amino acids from the position with the highest RF score (26th) to the fourth highest RF score (191 st) (Table 1). All five ML experiments showed high accuracy for classification of field PRRSV strains except one experiment using only 26th amino acid position (Figure 5). In the four experiments with 2 or more than 2 amino acid positions (2/3/4/200), all four ML approaches showed ~100% accuracy in terms of AUC, precision, recall, and f1-score (Table 2). However, in the experiment using one amino acid sequence with the highest importance score, 26th amino acid position, the accuracy decreased drastically to ~80% in all ML four methods. KNN showed a high variability in accuracy but other three ML approaches had relatively low variability. In the subsequent experiment adding one additional amino acid sequence with the second highest importance score, 170th position, all ML four methods showed very high accuracy of classification, as the experiment using all 200 amino acid positions did. Four ML algorithms classified field PRRSV strains in very short time consumption (<2.5 s). RF showed consistently short time consumption even with the changes in the number of amino acid sequences used. However, SVM and KNN required higher time consumption when they worked with all 200 amino acid sequences. MLP showed high variability in the time consumption with consistency as the number of used positions changed (Figure 5).

**Table 2:** The class-wise averaged approximated precision/recall/f1-score values for the corresponding four machine learning (ML) algorithm by five experiments.

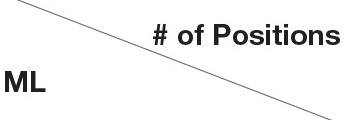	**200**	**4**	**3**	**2**	**1**
RF	0.99/0.99/0.99	0.99/0.99/0.99	0.99/0.99/0.99	0.99/0.99/0.99	0.75/0.85/0.79
SVM	0.99/0.99/0.99	0.99/0.99/0.99	0.99/0.99/0.99	0.99/0.99/0.99	0.75/0.85/0.79
KNN	0.99/0.99/0.99	0.99/0.99/0.99	0.99/0.99/0.99	0.98/0.98/0.98	0.73/0.83/0.77
CNN	0.99/0.99/0.99	0.99/0.99/0.99	0.99/0.99/0.99	0.99/0.98/0.98	0.73/0.83/0.77

## Discussion

The present study demonstrated that ML algorithms enabled to classify US field PRRSV strains into four clades accurately using five amino acid scores transformed from the ORF5 gene sequences with a short time consumption. Furthermore, one of four ML algorithms, specifically RF, was used to detect key amino acid positions potentially associated with biological characteristics of PRRSV strains.

In the present study, all four ML approaches accurately classified four clades even using small genetic information. Although each field PRRSV strain involved high-dimensional genome data including 1,000 features (5 scores × 200 amino acids), PCA visualization depicted that the genetic difference of field PRRSV strains between L5 and three L1 clades were distinctly distinguished. However, the genetic contiguity among L1A, L1B, and L1C clades posed a challenge for the classification of field PRRSV strains. In the classification using one amino acid sequence in 26th position with the highest RF importance score, all ML approaches showed fairly high AUC value (>0.79) and, RF, SVM and MLP classified field PRRSV strains stably comparing to KNN. Considering the significant heterogeneity of amino acid composition, the 26th position played a key role as a classifier between L5 and three L1A clades which constituted 83.9% of our PRRSV samples. Interestingly, after we additionally included one amino acid sequence in the 170th position, all four ML algorithms showed stable and very high accuracy for the classification (AUC > 0.99). Although the 170th amino acid position showed the homogeneity between L5 and L1A, this position showed significant heterogeneity among L1A, L1B, and L1C clades. Consequently, the combination of 26th and 170th amino acid sequences provided sufficient information for all ML approaches to identify the best-fit decision boundaries of classification among four clades. In the perspective of accuracy of classification, any of four ML approaches outperformed the RFLP typing. Specifically, considering very high stability of classification accuracy using all 200 amino acid sequences, RF might be a prioritized option to handle the high-dimensional genome data because RF, an ensemble ML algorithm, builds sufficiently a large number of decision trees and minimizes overfitting.

ML algorithms also have a great benefit in the time consumption compared to the phylogeny estimation. Generally, the classification using the phylogenetic analysis of infectious pathogen based on the large number of genome sequences requires high computational power and subsequent time consumption because the phylogeny estimation searches the unrooted phylogeny with the highest likelihood among possible unrooted phylogenies and the number of unrooted phylogenies gets exponentially increased by the number of sequences. However, all four ML approaches requires a very short amount of time for model training and classification of test data even with very large number of PRRSV sequences (<2.5 s). Specifically, RF and MLP showed a high consistency in the time consumption regardless of the number of features. Even with the small number of features, RF requires require a fair amount of time to generate large number of decision trees for stable model training. MLP also needs many computational steps to catch underlying characteristics of the data. However, considering the consistency of short and constant time consumption, RF and MLP could be well-adapted for the classification of the large genome data with high complexity rather than SVM and KNN.

The RF algorithm was used to detect key amino acid substitutions potentially associated with the biochemical characteristics of PRRSV. The 26th and 137th amino acid positions had high importance score with significant heterogeneity between L5 and three L1 clades. The 26th position of ORF5 gene showed the highest importance score and located in one of two cleavage sites in the decoying epitope of the GP5 protein (29). A previous study found that the amino acid substitution in the 26th position influenced on the host antibody response against PRRSV infection and characterized the infectivity of a PRRSV strain (30). In the 137th position with the third highest importance score, all L5 clade strains had Alanine and three L1 clade strains substituted to Serine. Alaine in the 137th position of ORF5 gene is generally monitored as a marker of Ingelvac PRRS Type 2 MLV in the L5 (Boehringer Ingelheim Vetmedica Inc., St. Joseph, Missouri, USA) because the substitution of Alaine to Serine in the 137th position of ORF5 gene considerably reduced the susceptibility of viral neutralization against VR2332 anti-serum, the reference strain of Ingelvac MLV (6, 23).

Although all four ML approaches showed very high accuracies in classification of field PRRSV strains, strong genetic homogeneity within clades comparing to heterogeneity among clades possibly inflated the accuracy in this study. The present study observed significant genetic heterogeneity among four clades of PRRSV strains, especially in the key 4 amino acid positions of ORF5 gene. Consequently, all four ML approaches led to nearly perfect accuracy for the classification even including two key amino acid sequences. It implies that our ML approaches potentially showed lower accuracy and longer time consumption in the multi-class classification with high complexity genome data. In the future research, we will explore ML approaches more complex classification using larger genetic information such as prediction of multiple phenotypic and antigenic characteristics classification to evaluate the accuracy and time consumption of ML approaches and the detection of key substitutions related with unique biological characteristics by each clades of field PRRSV strains.

In the modern livestock industry, genome sequencing enables to obtain high-quality and large genetic information of infectious pathogen and is widely applied to the genome-based diagnostics of infectious pathogen. This study exemplified the use of the high-quality genetic information for the classification of phenotypic characteristics of infectious pathogen. Once ML algorithms were sufficiently trained for classification, all ML algorithms accurately classified the genetic characteristics in a very short time and detected key amino acid sites, specifically for the rapid vaccine lineage selection based on genetic relatedness at a pig farm level (e.g., Prevacent® for lineage 1 and Ingelvac for lineage 5). We believe that our ML approaches using amino acid score for the classification of field PRRSV strains can be applied as a powerful tool in the digitalized surveillance system considering its very short time consumption and high accuracy. Furthermore, the use of ML approaches coupled with genetic information as we presented may inform decision makers in the US pig industry to have better understanding of PRRSV evolution and transmission dynamics and establish cost-effective control and preventive measures of PRRSV using MLV at farm or production system level.

## Conclusion

This study proposed the use of ML algorithms for classification of field PRRSV strains into four clades and detection of the key amino acid substitutions in ORF5 gene. Our ML approaches showed very high accuracy and short time consumption comparing to conventional approaches of PRRSV classification. We believe that our ML approaches based on amino acid score could be a powerful alternative to handle large genome dataset in real time or semi-real time to classify field PRRSV strains as well as other infectious pathogens and support decision-making or design more timely surveillance or intervention strategies.

## Data Availability Statement

The raw data supporting the conclusions of this article will be made available by the authors, without undue reservation.

## Author Contributions

JK and KL designed the study. JK developed the Python ML codes for experiments, carried out the analysis on experimental results, and wrote the draft of the manuscript. BM-L and KL collected, cleaned, and verified the data. RR contributed to writing background part and analysis the results. SR and XL helped to justify methodologies in this study and to edit method explanation part. All authors participated in the discussion and interpretation of the results, read, edited, and approved the final manuscript.

## Conflict of Interest

The authors declare that the research was conducted in the absence of any commercial or financial relationships that could be construed as a potential conflict of interest.

## Publisher's Note

All claims expressed in this article are solely those of the authors and do not necessarily represent those of their affiliated organizations, or those of the publisher, the editors and the reviewers. Any product that may be evaluated in this article, or claim that may be made by its manufacturer, is not guaranteed or endorsed by the publisher.
