# Supplementary material for: Applications of Machine Learning for the Classification of Porcine Reproductive and Respiratory Syndrome Virus Sublineages Using Amino Acid Scores of ORF5 Gene
Source: Front Vet Sci. 2021 Jul 23;8:683134. doi: 10.3389/fvets.2021.683134 (PMC8345883; doi:10.3389/fvets.2021.683134)
Supplement: Supplementary file 1 [file Table_1.pdf]

Supplement 1. The classification of 1931 field PRRSV strains into four clades (L1A, L1B, L1C and L5 clade) using the Restriction fragment length polymorphism (RFLP) analysis of Open reading frame 5 (ORF5) gene.

RFLP Type	L5 clade (n = 438)	L1A clade (n = 1,225)	L1B clade (n = 69)	L1C clade (n = 199)	Subtotal
1-1-1				1	1
1-1-2			30		30
1-10-2		1			1
1-10-4		9			9
1-12-4		3		4	7
1-16-2		1			1
1-16-4		6		1	7
1-18-2			2		2
1-18-3			1		1
1-19-4			3		3
1-2-2				2	2
1-2-4				11	11
1-21-4		5			5
1-24-2			2		2
1-26-2			28		28
1-26-4			2		2
1-3-1		1			1
1-3-2			1	1	2
1-3-3		1			1
1-3-4		8		1	9
1-30-4		1			1
1-33-3		3			3
1-33-4		1			1
1-4-1		6			6
1-4-3		1		6	7
1-4-4		35		67	102
1-45-4		1			1
1-5-2	10				10
1-6-2	1				1
1-6-4		3			3
1-7-2		15			15
1-7-4		297		1	298

1-8-1		1			1
1-8-2		10			10
1-8-3		27		3	30
1-8-4		788		100	888
1-8-8		1			1
1-45-4				1	1
2-1-2	9				9
2-5-1	3				3
2-5-2	410				410
2-5-4	2				2
2-6-2	3				3
**Total**	**438**	**1225**	**69**	**199**	**1931**
